# Long-Term Effects of Annual Intensive Rehabilitation in Patients with Hereditary Pure Cerebellar Ataxia: A 7-year Follow-up Study

**DOI:** 10.1007/s12311-025-01899-8

**Published:** 2025-09-04

**Authors:** Kyota Bando, Yuki Kondo, Yosuke Ariake, Taro Kato, Mari S. Oba, Takatoshi Hara, Yuji Takahashi

**Affiliations:** 1 Department of Rehabilitation, National Center Hospital, National Center of Neurology and Psychiatry, Tokyo, Japan; 2 Department of Science and Engineering, Kanto Gakuin University, Tokyo, Japan; 3 Department of Clinical Data Science, Clinical Research & Education Promotion Division, National Center of Neurology and Psychiatry, Tokyo, Japan; 4 Department of Neurology, National Center Hospital, National Center of Neurology and Psychiatry, Tokyo, Japan; 5 Department of Neurology, National Center of Neurology and Psychiatry, 4-1-1 Ogawahigashi-cho, Kodaira-shi, Tokyo, 187-8551 Japan

**Keywords:** Spinocerebellar ataxia, Rehabilitation, Postural balance, Ataxia, Longitudinal studies

## Abstract

Although intensive rehabilitation has achieved short-term benefits in patients with spinocerebellar degeneration, long-term outcomes of periodic intervention remain unclear, particularly in patients with pure spinocerebellar ataxia types 6 (SCA6) and 31 (SCA31). To investigate the longitudinal effects of annual intensive rehabilitation on ataxic symptoms and balance function in patients with pure cerebellar type SCA6 and SCA31. Seven patients with genetically confirmed SCA6 or SCA31 participated in annual 4-week intensive rehabilitation programmes. Each programme consisted of daily physical therapy, occupational/speech therapy, and self-directed balance training. The participants were assessed annually at pre-intervention, post-intervention, and the 6-month follow-up using the Scale for the Assessment and Rating of Ataxia (SARA) and Balance Evaluation Systems Test (BESTest). Changes were analysed using linear mixed-effect models. SARA scores were stable, indicating slower progression than the expected natural history, through year 6, with significant improvement observed post-intervention in year 2 (*p* = 0.04). Significant deterioration occurred at year 7 based on pre-intervention scores (*p* = 0.01), suggesting prolonged sustained benefits for coordination. The BESTest scores revealed an earlier decline, with significant deterioration from year 3 (*p* = 0.04), which progressed until year 7 (*p* < 0.01). Annual intensive rehabilitation effectively slowed the progression of ataxic symptoms (SARA) for up to six years, while balance function (BESTest) showed a significant decline from the third year. These findings indicate that an annual rehabilitation schedule is valuable for maintaining coordination but may be insufficient to prevent the progressive decline of balance function in patients with pure cerebellar ataxia.

**Supplementary Information:**

The online version contains supplementary material available at 10.1007/s12311-025-01899-8.

## Introduction

Spinocerebellar degeneration (SCD) encompasses a group of progressive neurodegenerative disorders that primarily affect the cerebellum [1]. Although hereditary SCDs comprise multiple subtypes with diverse clinical presentations based on their pathophysiology and causative genes, they share common cerebellar ataxia symptoms, including impaired coordination, intention tremor, dysarthria, and oculomotor abnormalities [2].

Spinocerebellar ataxia types 6 (SCA6) and 31 (SCA31) are classified as pure cerebellar types, characterised by the predominant degeneration of Purkinje cells in the cerebellar cortex [3–5]. These subtypes typically present with coordination deficits and intention tremor, followed by progressive deterioration of balance function. Pure cerebellar types generally exhibit slower disease progression than that observed with other genetic variants, necessitating treatment interventions with considerations of long-term efficacy [6].

Recent studies have suggested that intensive rehabilitation may lead to short-term improvements in coordination deficits and gait ability [7–9]. Intensive and repetitive motor learning programmes are considered practical approaches, potentially promoting neuroplasticity at cerebellar and cerebral cortical levels [10–12]. However, studies investigating the long-term effects of continuous intervention over multiple years remain scarce, with no long-term intervention studies specifically targeting patients with SCA6 and SCA31 types [13, 14].

Furthermore, understanding which symptoms show sustained improvements following intensive rehabilitation is crucial. Aerobic exercise using ergometers may improve coordination deficits, whereas balance training may be more effective for improving balance function than for improving coordination deficits [15]. The cerebellar cortex exhibits functional segregation, with the vermis primarily controlling standing balance and the intermediate portion regulating limb coordination [16, 17]. Thus, the effects of long-term interventions should be evaluated from perspectives of coordination and balance function.

In this study, we conducted annual intensive rehabilitation sessions for up to 7 years in patients with SCA6 and SCA31. We assessed yearly progression using the Scale for the Assessment and Rating of Ataxia (SARA) for ataxic symptoms and Balance Evaluation Systems Test (BESTest) for balance function [18, 19]. Our objective was to elucidate the differential progression patterns of ataxic symptoms versus balance function and to determine the potential role of annual interventions. The findings of this study may contribute to establishing comprehensive long-term rehabilitation strategies for pure cerebellar-type ataxia.

## Patients and Methods

### Study Design and Participants

This retrospective cohort study was conducted in accordance with the Declaration of Helsinki and was approved by the Ethics Committee of the National Center of Neurology and Psychiatry (approval number A2023-104). All participants provided informed consent after receiving a verbal explanation of the purpose, methods, anticipated benefits, and potential risks of the study.

We retrospectively screened 160 patient records of patients with SCD who underwent an intensive rehabilitation programme at our centre between June 2015 and April 2021. Eligibility for the rehabilitation programme required that participants could walk independently (with or without assistive devices) and preserve community living with minimal or no assistance.

From these records, we selected patients who met the following two criteria for the analysis in this study: (1) a genetic diagnosis of SCA6 or SCA31 and (2) participation in the annual 4-week intensive rehabilitation programme at least three times. This criterion was established because a minimum of three assessment points is necessary to reliably analyse a longitudinal trajectory. This threshold represents an optimal balance between ensuring the methodological validity for trend analysis and maintaining sufficient number of participants from our retrospective cohort for statistical evaluation. This resulted in a final cohort of seven patients. All the seven participants included in the analysis completed the full 4-week programme annually throughout their respective follow-up periods. The demographic and clinical characteristics of the participants are presented in Table 1. The cohort included two patients with SCA6 and five with SCA31. The mean age of onset was 53.9 years and at study entry was 66.4 years.

**Table 1:** Demographic and clinical characteristics of study participants at baseline

ID	Disease Type	Sex	Age at study entry	Age at Onset	Base SARA sum score	Base BESTest sum score
1	SCA31	Male	70	63	9	93
2	SCA31	Female	64	52	6.5	89
3	SCA6	Male	70	60	14.5	74
4	SCA31	Female	78	65	7.5	87
5	SCA31	Male	54	48	5.5	93
6	SCA6	Male	71	46	4.5	96
7	SCA31	Male	58	43	12.5	69

Age at study entry refers to the participant’s age at the start of the rehabilitation program. Age at onset represents the age at which the patient or family members noticed the first symptoms. Base SARA and BESTest scores were obtained at the initial assessment before the first rehabilitation intervention

Abbreviations: *SCA6* spinocerebellar ataxia type 6, *SCA31* spinocerebellar ataxia type 31, *SARA* Scale for the Assessment and Rating of Ataxia (score range: 0–40, higher scores indicate more significant impairment), *BESTest* Balance Evaluation Systems Test (score range: 0–108, higher scores indicate better balance function)

### Rehabilitation Intervention Programme

Participants underwent rehabilitation 5 days per week over a 4-week period while hospitalised. The daily programme consisted of three 1-hour sessions, consisting of individualised physical therapy, occupational or speech therapy, and self-directed balance training sessions. The physical therapy sessions incorporated balance training and ergometer exercises for lower limb ataxia, following the rehabilitation guidelines established by the Research Committee on Ataxia of the Ministry of Health, Labour and Welfare of Japan (http://ataxia.umin.ne.jp/rehabilitation/). Occupational therapy focused on fine motor skills, whereas speech therapy provided articulation training for patients with dysarthria. Self-directed training sessions included safety-monitored mat exercises, kneeling, and quadruped positions, as prescribed by physical therapists for individual practice.

### Outcome Measures

Assessments were conducted annually at three-time points: pre-intervention (pre), post-intervention (post), and at 6 months post-intervention (follow-up). The primary outcome measure was the SARA scale, with scores ranging from 0 to 40 points and higher scores indicating greater severity of ataxia [20]. The balance function was evaluated using the BESTest, which was scored from 0 to 108 points, with higher scores indicating better balance function [21]. To minimise measurement bias, evaluations were conducted by physical therapists who were not involved in the intervention and had received comprehensive training in assessment procedures.

### Statistical Analysis

To analyse the yearly changes in SARA and BESTest scores, we employed a linear mixed-effect model (LMM) using R (v.4.0.2; R Foundation for Statistical Computing, Vienna, Austria) with the lme4 and lmerTest packages [22]. The dependent variables were changes in SARA and BESTest scores from baseline (year 1), fixed effects for evaluation year and disease type (SCA31 and SCA6), and random effects for individual ID. Analyses were conducted separately for pre-intervention (to investigate yearly changes), for post-intervention (to assess intervention effects), and at the follow-up (to evaluate effect retention) time points.

Given the small sample size (*n* = 7), we limited the number of fixed effects in the model to avoid potential overfitting; therefore, other potential covariates, such as age at study entry and disease duration, were not included in the final model. For the sensitivity analysis, we re-analysed the models, replacing the disease type covariate with age at study entry or disease duration, and confirmed that the primary findings at all evaluation time points (admission, discharge, and 6-month follow-up) were unchanged (Supplementary Tables 1–3).

## Results

### Changes in SARA Scores

**Fig. 1:**
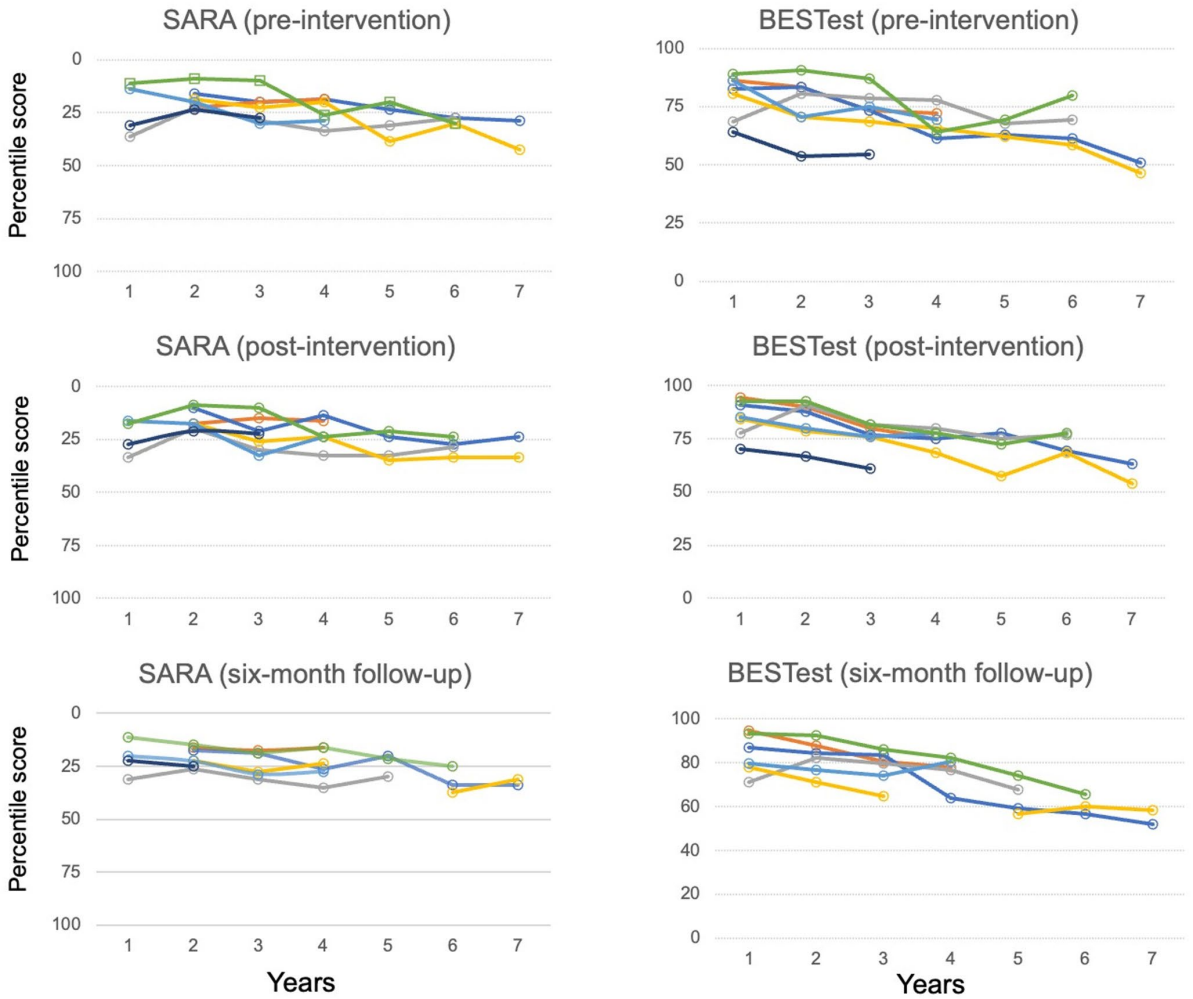
Individual longitudinal changes in SARA and BESTest scores over the 7-year follow-up. Note: Values are shown as percentile-transformed scores comparing SARA and BESTest scores (SARA: 0–40 points; BESTest: 0–108 points). The y-axis for SARA scores is inverted because lower scores indicate better function, whereas higher BESTest scores indicate better balance performance. Graphs should be interpreted such that a downward trend indicates worsening of symptoms. SARA, Scale for the Assessment and Rating of Ataxia; BESTest, Balance Evaluation Systems Test

The linear mixed model analysis of SARA scores revealed several significant temporal changes (Fig. 1; Table 2). Analysis of yearly changes from baseline pre-intervention scores showed estimated changes of −1.34 (95% confidence intervals [CI] [−4.24, 1.56], *p* = 0.37) at year 2 and 0.16 (95% CI [−2.74, 3.06], *p* = 0.92) at year 3. The 95% CI continued to cross zero through year 6. A significant increase of 5.78 (95% CI [1.54, 10.03], *p* = 0.01) was observed at year 7.

**Table 2:** Linear mixed model analysis of changes in SARA score from baseline over the 7-year follow-up period

A. Pre-intervention assessment				
Factor	Estimate	SE	95% CI	P-value
year2	−1.34	1.48	[−4.24, 1.56]	0.37
year3	0.16	1.48	[−2.74, 3.06]	0.92
year4	1.12	1.55	[−1.91, 4.15]	0.48
year5	2.85	1.71	[−0.49, 6.2]	0.11
year6	2.98	1.71	[−0.37, 6.32]	0.09
year7	5.78	2.16	[1.54, 10.03]	0.01**
B. Post-intervention assessment				
Factor	Estimate	SE	95% CI	P-value
year2	−2.68	1.19	[−5.01, −0.35]	0.04*
year3	−0.11	1.19	[−2.44, 2.22]	0.93
year4	−0.02	1.25	[−2.47, 2.42]	0.99
year5	2.19	1.38	[−0.51, 4.89]	0.13
year6	2.31	1.38	[−0.39, 5.02]	0.11
year7	2.52	1.75	[−0.91, 5.95]	0.16
C. 6-Month follow-up assessment				
Factor	Estimate	SE	95% CI	P-value
year2	0.47	0.72	[−0.95, 1.89]	0.52
year3	1.90	0.77	[0.39, 3.41]	0.02*
year4	2.07	0.77	[0.55, 3.58]	0.02*
year5	1.73	0.90	[−0.04, 3.51]	0.07
year6	5.97	0.94	[4.13, 7.81]	< 0.01**
year7	5.33	1.09	[3.2, 7.46]	< 0.01**

Estimates represent changes from baseline (year 1) scores. Positive values indicate worsening, and negative values indicate improvement. Analysis was performed using a linear mixed-effects model with fixed effects for year and disease type and random effects for individual participants. *P < 0.05, **P < 0.01

Abbreviations: *SARA* Scale for the Assessment and Rating of Ataxia, *SE* Standard Error, *df* degrees of freedom, *CI* Confidence Interval

Post-intervention evaluation revealed significant improvement at year 2 (−2.68, 95% CI [−5.01, −0.35], *p* = 0.04). The estimated change in year 3 was − 0.11; at year 4, it was − 0.02. Although estimates showed an increasing trend from year 5 onward, all 95% CI values crossed zero.

At the 6-month follow-up, despite no significant differences at year 2 (0.47, 95% CI [−0.95, 1.89], *p* = 0.52), significant increases were observed in year 3 (1.90, 95% CI [0.39, 3.41], *p* = 0.02) and year 4 (2.07, 95% CI [0.55, 3.58], *p* = 0.02), with evidence of more pronounced deterioration in years 6 to 7.

### Changes in BESTest Scores

The analysis of BESTest scores demonstrated a more pronounced pattern of decline (Fig. 1; Table 3). Pre-intervention BESTest scores showed no significant differences at year 2 (−3.71, 95% CI [−10.24, 2.81], *p* = 0.28), but a significant decline was observed from year 3 (−7.14, 95% CI [−13.66, −0.62], *p* = 0.04) onwards, reaching − 32.04 (95% CI [−42.30, −21.79], *p* < 0.01) by year 7.

**Table 3:** Linear mixed model analysis of changes in the bestest score from baseline over the 7-year follow-up period

A. Pre-intervention assessment				
Factor	Estimate	SE	95% CI	P-value
year2	−3.71	3.33	[−10.24, 2.81]	0.28
year3	−7.14	3.33	[−13.66, −0.62]	0.04*
year4	−14.00	3.50	[−21.81, −8.09]	< 0.01**
year5	−18.03	4.03	[−25.93, −10.14]	< 0.01**
year6	−16.28	4.03	[−24.18, −8.39]	< 0.01**
year7	−32.04	5.23	[−42.3, −21.79]	< 0.01**
B. Post-intervention assessment				
Factor	Estimate	SE	95% CI	P-value
year2	−1.43	2.36	[−6.06, 3.2]	0.55
year3	−9.71	2.36	[−14.35, −5.08]	< 0.01**
year4	−12.75	2.49	[−17.62, −7.87]	< 0.01**
year5	−18.21	2.87	[−23.82, −12.59]	< 0.01**
year6	−15.46	2.87	[−21.07, −9.84]	< 0.01**
year7	−28.49	3.72	[−35.78, −21.19]	< 0.01**
C. 6-Month follow-up assessment				
Factor	Estimate	SE	95% CI	P-value
year2	−0.33	3.61	[−7.41, 6.74]	0.93
year3	−5.16	3.61	[−12.29, 1.91]	0.17
year4	−8.50	3.82	[−16.01, −1]	0.04*
year5	−19.46	4.15	[−27.59, −11.34]	< 0.01**
year6	−23.32	4.59	[−32.32, −14.32]	< 0.01**
year7	−24.00	5.38	[−35.34, −14.49]	< 0.01**

Estimates represent changes from baseline (year 1) scores. Negative values indicate deterioration in balance function, as BESTest scores range from 0 to 108, with higher scores indicating better balance function. Analysis was performed using a linear mixed-effects model with fixed effects for year and disease type and random effects for individual participants. *P < 0.05, **P < 0.01

Abbreviations: *BESTest* Balance Evaluation Systems Test, *SE* Standard Error, *df* degrees of freedom, *CI* Confidence Interval

Post-intervention assessments demonstrated a consistent significant decline from year 3 onward, with follow-up evaluations showing significant balance deterioration from year 4 (−8.50, 95% CI [−16.01, −1.00], *p* = 0.04) onwards. Notably, years 5 to 7 showed decreases exceeding 19 points in BESTest scores, suggesting a relatively faster progression of balance dysfunction compared to that observed with the SARA scores.

## Discussion

To the best of our knowledge this study represents the first longitudinal investigation of annual intensive rehabilitation programmes in patients with hereditary pure cerebellar ataxia (SCA6 and SCA31) over an extended follow-up period of up to 7 years. The study examined ataxic symptoms (SARA) and balance function (BESTest). The findings indicate that annual intensive rehabilitation can slow the progression of coordination deficits, as indicated by SARA, over several years, providing valuable long-term benefits. However, balance function, assessed by the BESTest, showed a different trajectory, highlighting potential areas for refinement of long-term management strategies.

Examining the effects on coordination, assessed using the SARA scale, the primary goal of rehabilitation intervention in patients with SCD is to minimise disease progression [23]. The natural history of SARA scores in patients with SCA6 and SCA31 typically presents an average annual deterioration of 0.8 points [24, 25]. Our results demonstrated that the pre-intervention SARA estimates remained relatively stable compared with the reported natural history through year 6, suggesting that the annual intervention may have contributed to slowing disease progression in terms of coordination. Although year 7 showed a significant increase in the pre-intervention score compared to baseline, this finding may be influenced by the smaller sample size in the final year. Intervention effects, assessed post-intervention, showed significant improvement in year 2 (−2.68, *p* = 0.04) and remained relatively stable through years 3 and 4 (−0.11 and − 0.02, respectively), with a trend towards deterioration from year 5 onwards. However, the 95% CIs for post-intervention change continued to cross zero through year 7, which indicated that the annual rehabilitation programme had a lasting, albeit diminishing, effect on coordination deficits even after 7 years. One contributing factor may have been our programme’s emphasis on aerobic exercise using ergometers, which targets limb coordination and is effective in improving SARA scores [15, 26]. These findings underscore the long-term value of periodic intensive rehabilitation for maintaining coordination.

To better contextualise the clinical relevance of these SARA score changes, our findings can be compared with previously reported values for the smallest detectable change (SDC). A prior study established an SDC of approximately 3.5 points for individual patients and 0.3 points for group-level analysis [27]. Our statistically significant findings, such as the post-intervention improvement in SARA score in year 2 (−2.68 points) and the deterioration in year 7 (+ 5.79 points), both exceeded the group SDC threshold, supporting the finding that these were true changes for the cohort. However, this comparison should be interpreted with caution, as the cohort in that reference study was heterogeneous and included a large proportion of patients with other SCA genotypes (such as SCA1, SCA2, and SCA3), whose disease progression may differ from the pure cerebellar types in our study. Furthermore, these SDC values are based on distribution methods, and future research should focus on establishing an anchor-based minimal clinically important difference (MCID) to better record changes that are meaningful to patients.

The retention effect at the 6-month follow-up showed a decreasing trend over the years, becoming significantly worse than baseline from year 3 onwards. The cerebellum plays a crucial role in motor learning [28, 29], and intensive motor programmes in patients with SCD induce plastic changes in the cerebellum and in functionally connected cerebral cortical areas [11, 12]. However, SCD causes progressive reduction in cerebellar cortical volume over time [30, 31], and motor learning capacity reportedly declines with increasing disease duration [32]. These factors likely contributed to the observed decline in the retention of intervention effects, suggesting that although short-term motor learning remains possible, long-term retention capacity may be compromised by disease progression. This highlights the importance of the annual repetition of the intensive program to reinforce benefits.

A key finding of this long-term study is the positive impact of annual intensive rehabilitation on coordination function, as assessed by the SARA scale. Our results suggest that this periodic intervention helped to stabilise SARA scores compared with the expected natural history for up to six years, demonstrating valuable long-term benefits in managing core ataxic symptoms in patients with SCA6 and SCA31. This underscores the potential of structured, repeated rehabilitation efforts to mitigate disease progression in specific functional domains.

In contrast, balance function, measured by the BESTest, showed a significant decline starting from years 3 to 4, a trend observed across all assessment time points. This earlier deterioration highlights a divergence in the long-term response between coordination and balance under this specific annual intervention protocol. Several factors may contribute to this difference. Balance control relies on a more complex integration of cerebellar functions with multiple sensory inputs (visual, vestibular, and somatosensory) and motor outputs [33–38], potentially making it more vulnerable to overall disease progression or less amenable to improvement due to the specific exercises emphasised in our program (e.g. ergometer cycling targeting limb coordination, likely benefiting SARA scores more directly [15, 26]). Furthermore, the annual 4-week format, albeit effective for coordination for several years, might be insufficient in frequency or duration to fully counteract the decline in the multifaceted demands of balance control over such an extended period.

Although the decline in BESTest scores indicates a need to explore complementary strategies specifically targeting balance [39, 40], it does not diminish the significant finding regarding SARA scores. The sustained relative stability in coordination function over multiple years provides strong evidence for the clinical utility of annual intensive rehabilitation in the long-term management of pure cerebellar ataxia. This periodic approach appears valuable for preserving limb coordination, a critical aspect of daily function for these patients.

Future research, including controlled trials with a larger patient cohort, is warranted to further delineate the effects of intensive rehabilitation on different functional domains and investigate optimal rehabilitation frequencies, durations, and components aimed at exploring integrated approaches to better address both coordination and the challenging progressive decline in balance. Furthermore, establishing a population-specific, anchor-based MCID for pure cerebellar ataxia is crucial for a more precise interpretation of clinical relevance.

### Limitations

This study has some limitations. First, as a retrospective study, complete standardisation of rehabilitation content and evaluation periods was impossible. Second, the small sample size of seven participants does not allow generalisation of the results. Third, the extent of adherence to self-directed training was not quantitatively monitored, which could have introduced unmeasured variability in the outcomes. Additionally, the absence of a control group prevents direct comparison with the natural history of the condition, and the linear mixed model analysis may not fully capture potential non-linear disease progression patterns. Finally, this study did not include patient-reported outcome measures, which are important for fully understanding the clinical impact of the intervention.

## Conclusions

This 7-year longitudinal study demonstrated that an annual 4-week intensive rehabilitation has significant long-term benefits for managing coordination deficits in patients with pure cerebellar ataxia. SARA scores remained relatively stable compared with the expected natural history for several years, while balance function, assessed using BESTest, showed an earlier and more progressive decline. These findings support the clinical utility of this periodic intervention for preserving coordination. However, they also highlight that maintaining balance is more challenging and may require supplementary or more frequent therapeutic approaches, warranting future research into optimised and comprehensive management strategies.

## Supplementary Information

Below is the link to the electronic supplementary material.

Supplementary Material 1 (DOCX 18.2 KB)

Supplementary Material 2 (DOCX 16.9 KB)

Supplementary Material 3 (DOCX 16.8 KB)

## Data Availability

No datasets were generated or analysed during the current study.
