# Supplementary material for: Long-Term Effects of Annual Intensive Rehabilitation in Patients with Hereditary Pure Cerebellar Ataxia: A 7-year Follow-up Study
Source: Cerebellum. 2025 Sep 4;24(5):150. doi: 10.1007/s12311-025-01899-8 (PMC12411578; doi:10.1007/s12311-025-01899-8)
Supplement: Supplementary file 1 — Supplementary Material 1 (DOCX 18.2 KB) [file 12311_2025_1899_MOESM1_ESM.docx]

| Fixed Effect (Term) | Model 1 (disease) | Model 2 (Age) | Model 3 (Duration) |
| --- | --- | --- | --- |
|  | Estimate (SE) | Estimate (SE) | Estimate (SE) |
| year2 | -1.34 (1.48) | -1.33 (1.20) | -1.31 (1.21) |
| year3 | 0.16 (1.48) | 0.15 (1.49) | 0.16 (1.49) |
| year4 | 1.12 (1.55) | 1.35 (1.49) | 1.37 (1.49) |
| year5 | 2.85 (1.71) | 2.87 (1.60) | 2.81 (1.60) |
| year6 | 2.98 (1.71) | 2.57 (1.76) | 2.53 (1.76) |
| year7 | 5.78 (2.16)** | 5.84 (2.17)** | 5.73 (2.17)* |

Supplementary Table 1. Sensitivity analysis for changes in SARA and BESTest scores at admission (pre-intervention)

A) SARA score at admission

B) BESTest score at admission

| Fixed Effect (Term) | Model 1 (disease) | Model 2 (Age) | Model 3 (Duration) |
| --- | --- | --- | --- |
|  | Estimate (SE) | Estimate (SE) | Estimate (SE) |
| year2 | -3.71 (3.33) | -3.97 (3.34) | -3.93 (3.36) |
| year3 | -7.14 (3.33)* | -7.23 (3.33)* | -7.44 (3.34)* |
| year4 | -14.00 (3.50)** | -12.81 (4.50)* | -12.91 (4.51)* |
| year5 | -18.03 (4.03)** | -17.55 (4.68)** | -17.65 (4.69)** |
| year6 | -16.28 (4.03)** | -18.99 (5.22)** | -19.06 (5.23)** |
| year7 | -32.04 (5.23)** | -31.97 (5.24)** | -32.00 (5.25)** |

Note: All data is from the sensitivity analysis results. Model 1 is adjusted for disease type. Model 2 is adjusted for age at study entry. Model 3 is adjusted for disease duration. SE: Standard Error. *P < 0.05, **P < 0.01.
