# Supplementary material for: Long-Term Effects of Annual Intensive Rehabilitation in Patients with Hereditary Pure Cerebellar Ataxia: A 7-year Follow-up Study
Source: Cerebellum. 2025 Sep 4;24(5):150. doi: 10.1007/s12311-025-01899-8 (PMC12411578; doi:10.1007/s12311-025-01899-8)
Supplement: Supplementary file 2 — Supplementary Material 2 (DOCX 16.9 KB) [file 12311_2025_1899_MOESM2_ESM.docx]

Supplementary Table 2. Sensitivity analysis for changes in SARA and BESTest scores at discharge (post-intervention)

A) SARA score at discharge

| Fixed Effect (Term) | Model 1 (disease) | Model 2 (Age) | Model 3 (Duration) |
| --- | --- | --- | --- |
|  | Estimate (SE) | Estimate (SE) | Estimate (SE) |
| year2 | -2.68 (1.19)* | -2.69 (1.19)* | -2.65 (1.20)* |
| year3 | -0.11 (1.19) | -0.13 (1.47) | -0.12 (1.48) |
| year4 | -0.02 (1.25) | -0.02 (1.48) | 0.01 (1.48) |
| year5 | 2.19 (1.38) | 2.54 (1.48) | 2.39 (1.59) |
| year6 | 2.31 (1.38) | 2.41 (1.45) | 2.47 (1.75) |
| year7 | 2.52 (1.75) | 2.43 (1.16) | 2.53 (1.16) |

B) BESTest score at discharge

| Fixed Effect (Term) | Model 1 (disease) | Model 2 (Age) | Model 3 (Duration) |
| --- | --- | --- | --- |
|  | Estimate (SE) | Estimate (SE) | Estimate (SE) |
| year2 | -1.43 (2.36) | -1.56 (2.59) | -1.58 (2.21) |
| year3 | -9.71 (2.36)** | -7.86 (3.58)* | -8.03 (3.60)* |
| year4 | -12.75 (2.49)** | -12.78 (2.45)** | -12.84 (2.47)** |
| year5 | -18.21 (2.87)** | -17.76 (2.64)** | -17.84 (2.66)** |
| year6 | -15.46 (2.87)** | -16.06 (2.18)** | -15.12 (2.20)** |
| year7 | -28.49 (3.72)** | -27.86 (3.19)** | -28.89 (3.21)** |

Note: All data is from the sensitivity analysis results. Model 1 is adjusted for disease type. Model 2 is adjusted for age at study entry. Model 3 is adjusted for disease duration. SE: Standard Error. *P < 0.05, **P < 0.01.
