# Supplementary material for: Long-Term Effects of Annual Intensive Rehabilitation in Patients with Hereditary Pure Cerebellar Ataxia: A 7-year Follow-up Study
Source: Cerebellum. 2025 Sep 4;24(5):150. doi: 10.1007/s12311-025-01899-8 (PMC12411578; doi:10.1007/s12311-025-01899-8)
Supplement: Supplementary file 3 — Supplementary Material 3 (DOCX 16.8 KB) [file 12311_2025_1899_MOESM3_ESM.docx]

Supplementary Table 3. Sensitivity analysis for changes in SARA and BESTest scores at the 6-month follow-up

A) SARA score at the 6-month follow-up

| Fixed Effect (Term) | Model 1 (disease) | Model 2 (Age) | Model 3 (Duration) |
| --- | --- | --- | --- |
|  | Estimate (SE) | Estimate (SE) | Estimate (SE) |
| year2 | 0.47 (0.72) | 0.46 (0.72) | 0.48 (0.72) |
| year3 | 1.90 (0.77)* | 1.89 (0.77)* | 1.87 (0.77)* |
| year4 | 2.07 (0.77)* | 2.08 (0.77)* | 2.05 (0.78)* |
| year5 | 1.73 (0.90) | 1.83 (0.90) | 1.86 (0.90) |
| year6 | 5.97 (0.94)** | 5.32 (1.11)** | 5.29 (1.11)** |
| year7 | 5.33 (1.09)** | 5.30 (1.12)** | 5.21 (1.02)** |

B) BESTest score at the 6-month follow-up

| Fixed Effect (Term) | Model 1 (disease) | Model 2 (Age) | Model 3 (Duration) |
| --- | --- | --- | --- |
|  | Estimate (SE) | Estimate (SE) | Estimate (SE) |
| year2 | -0.33 (3.61) | -0.29 (3.59) | -0.31 (3.61) |
| year3 | -5.16 (3.61) | -5.28 (3.73) | -5.42 (3.74) |
| year4 | -8.50 (3.82)* | -8.45 (3.83)* | -8.50 (3.84)* |
| year5 | -19.46 (4.15)** | -19.67 (4.13)** | -19.75 (4.15)** |
| year6 | -23.32 (4.59)** | -24.00 (4.71)** | -23.06 (4.32)** |
| year7 | -24.00 (5.38)** | -23.76 (4.73)** | -23.78 (5.74)** |

Note: All data is from the sensitivity analysis results. Model 1 is adjusted for disease type. Model 2 is adjusted for age at study entry. Model 3 is adjusted for disease duration. SE: Standard Error. *P < 0.05, **P < 0.01.
